# The Impact of Anesthesia on Dermatological Outcomes: A Narrative Review

**DOI:** 10.7759/cureus.72321

**Published:** 2024-10-24

**Authors:** Rahib K Islam, Victoria T Tong, Cameron Robicheaux, Hayden Tageant, Christopher J Haas, Ryan J Kline, Kazi N Islam

**Affiliations:** 1 School of Medicine, Louisiana State University (LSU) Health Sciences Center New Orleans, New Orleans, USA; 2 School of Medicine, Louisiana State University (LSU) Health Sciences Center Shreveport, Shreveport, USA; 3 School of Medicine, Louisiana State University Health Sciences Center Shreveport, Shreveport, USA; 4 Dermatology, Louisiana State University (LSU) Health Sciences Center New Orleans, New Orleans, USA; 5 Anesthesiology, Louisiana State University (LSU) Health Sciences Center New Orleans, New Orleans, USA; 6 Agricultural Research Development Program, Central State University, Wilberforce, USA

**Keywords:** allergic reactions, anesthesia in dermatology, local anesthesia, pain management, topical anesthesia, wound healing

## Abstract

Anesthesia is an essential component of dermatologic procedures, influencing pain management and patient outcomes, including wound healing, infection control, and cosmetic appearance. This review examines the impact of various anesthetic techniques, topical, local, regional, and general, on dermatological outcomes. The findings reveal that while local anesthesia is preferred due to its efficacy and safety, specialized considerations are necessary for pediatric, geriatric, and high-risk patients. Anesthesia-related complications, such as allergic reactions, systemic toxicity, and delayed healing, require careful selection of agents and techniques. Innovations in anesthetic technology, including nanotechnology, microneedle patches, and cryoanesthesia, promise to improve pain management and minimize complications. Personalized anesthesia approaches, informed by genetic and proteomic analyses, offer the potential to optimize individual patient care. However, further research is needed to understand the long-term effects of anesthetic agents on wound healing and scarring, especially in patients with comorbidities. Overall, this review emphasizes the evolving role of anesthesia in dermatology and highlights the need for ongoing innovation to enhance patient care, minimize risks, and improve procedural outcomes.

## Introduction and background

Anesthesia is a cornerstone of modern dermatological practice, facilitating a wide range of procedures from simple excisions to more advanced interventions like laser treatments and Mohs micrographic surgery [1]. The role of anesthesia extends far beyond its traditional function of providing analgesia; it influences several critical aspects of dermatological outcomes, including wound healing, infection control, cosmetic appearance, and patient satisfaction [2]. As dermatologic procedures continue to evolve, the choice of anesthesia has become increasingly nuanced, potentially impacting the quality of care and the overall patient experience [3].

Different types of anesthesia, topical, local, regional, and general, are employed depending on the complexity of the procedure, patient health, and desired outcomes [4]. While local anesthesia is the most common in dermatology due to its efficacy and low risk, more complex cases may require regional blocks or general anesthesia [5]. Each technique has its own set of benefits and limitations, and depending on the type of anesthesia chosen for the procedure, this can significantly alter postoperative healing, affecting scar formation and cosmetic results [6,7].

Moreover, anesthesia-related complications such as allergic reactions, systemic toxicity, and delayed healing pose challenges that need to be addressed through careful selection of anesthetic agents and techniques [8]. Additionally, special populations, such as pediatric or geriatric patients and those with underlying comorbidities, present unique challenges that must be considered to optimize outcomes [9].

This review aims to comprehensively examine the impact of various anesthetic techniques on dermatologic outcomes. By delving into their effects on wound healing, infection rates, pain management, cosmetic results, and potential complications, this paper seeks to offer a broad perspective on the current state of anesthesia in dermatology. Furthermore, the review will highlight emerging trends, such as personalized anesthesia and minimally invasive techniques, while identifying areas where further research is needed. Ultimately, this discussion aims to equip dermatologists with the knowledge to make informed anesthetic choices that enhance patient outcomes and satisfaction.

## Review

Types and impact of outcomes regarding anesthesia utilized in dermatological procedures

Knowledge of the various types of anesthetics allows physicians, including dermatologists, to select the appropriate method and agent to keep their patients safe while obtaining optimal results. Topical anesthetics work by blocking the conduction of nerve impulses in sensory nerves through the inhibition of voltage-gated sodium channels in the neuronal membrane, essential for initiating and propagating action potentials. When applied, these anesthetics, such as lidocaine or benzocaine, penetrate the skin to reach sensory nerve endings. They diffuse through the neuronal cell membrane, binding to sodium channels in their inactivated state, thereby preventing sodium ions from entering the neuron. This blockage is crucial for nerve depolarization and the generation of action potentials. Without the influx of sodium, the neuron cannot reach the threshold potential necessary for initiating and transmitting nerve signals, resulting in localized numbness or analgesia. The effect of this blockade is reversible; once the anesthetic is metabolized or removed, normal nerve function resumes. Since these anesthetics primarily act on peripheral sensory nerves, they provide localized pain relief in the application area without affecting deeper tissues or having systemic effects, making them ideal for minor skin procedures and pain management in superficial skin lesions [10]. Due to their percutaneous application, topical anesthetics must penetrate the epidermis, which acts as a barrier to deeper layers such as the dermis, where the targeted nerves and blood vessels lie [11]. They are typically reserved for very superficial procedures [12]. Some of the most common topical anesthetic agents that penetrate intact skin include a eutectic mixture of local anesthetics (EMLA), ELA-Max, amethocaine, and lidocaine in acid mantle or velvachol. EMLA, containing lignocaine and prilocaine, is applied to the skin in a thick layer before covering the area with an occlusive dressing such as plastic wrap or Tegaderm to facilitate penetration [10]. It is recommended that EMLA be applied at least 1 hour before minor procedures [11]. EMLA reaches its peak effect at 2-3 hours, and the duration of its anesthetic effect can be influenced by the surface area covered; larger surface areas may enhance absorption and prolong its activity. Once removed, it continues to exert an anesthetic effect for 1-2 hours [10]. ELA-Max, which contains lidocaine, only requires 15-40 minutes following application to take effect [10]. Amethocaine, which is 4% tetracaine, also requires application under an occlusive dressing. Due to its lipophilic properties, Amethocaine has a faster onset of action, 40 minutes, and a duration of action of 4 hours, compared to EMLA [11]. Topical lidocaine does not penetrate the epidermis as readily as tetracaine and therefore requires higher concentrations (30-40%), such as those seen in lidocaine with acid mantle or velvachol used as a means for application [11].

Local anesthetics have a similar mechanism of causing analgesia by inhibiting sensory nerves from transmitting signals. However, they are typically injected into the tissue and anesthetize a larger surface area than topical agents [13]. The human body contains various types of nerve fibers, including C fibers, which are unmyelinated and transmit pain signals slowly, and A-delta fibers, which are myelinated and responsible for the rapid transmission of sharp, acute pain. Local anesthetic exerts most of its effect on these A-delta fibers, effectively blocking pain detection but potentially allowing a patient to continue to feel pressure and vibration, whereas C fibers are relatively resistant [12]. Selection of the appropriate local anesthetic largely depends on the goal of the procedure and physician preference, but knowledge of the various agents available is essential. The most common local anesthetic is 1% lidocaine with epinephrine in a concentration ratio of 1:200,000 because of its quick onset and excellent safety profile [12]. For a longer duration, etidocaine and bupivacaine are more likely to be appropriate. There are various techniques to consider when using local anesthetics, including infiltrative and tumescent techniques. Infiltrative anesthesia is typically administered at a depth of approximately 3 to 5 mm below the skin surface. This depth targets the dermis and superficial subcutaneous tissue, ensuring that the anesthetic reaches the nerve endings responsible for sensation in the area [12]. Then, the physician may progressively expand the anesthetized area by passing the needle through previously anesthetized tissue. This dramatically improves the patient’s comfort and tolerance of the procedure [13]. Tumescent local anesthesia is similar to infiltrative anesthesia, except it utilizes a high volume of diluted local anesthetic agent to inject the desired area [12]. Epinephrine may also be added to the injected solution, but this can increase the patient's uncomfortable burning sensation. To lessen the patient’s discomfort, sodium bicarbonate is also commonly added, with the proper ratio being 1 mL of sodium bicarbonate (8.4%) to 10 mL of local anesthetic solution [13].

Following the natural order of progression in the size of affected anesthetized areas, regional anesthesia involves anesthetizing a larger area of tissue or a peripheral sensory nerve supplying an anatomical body part [14]. Regional anesthesia targeting a peripheral sensory nerve, commonly referred to as a nerve block, is typically performed on the fingers and toes but can also apply to the head, neck, and penis in appropriate dermatologic cases [13,14]. Due to increased sensitivity in these areas, the fingers and face are commonly anesthetized using regional anesthesia to optimize patient comfort [15]. Regional anesthesia for the head and neck area has mainly been replaced with general anesthesia to avoid systemic toxicity, which can result relatively quickly in this region [15]. Familiarity with the anatomy is required to properly locate the peripheral nerve and anesthetize the desired area [14]. Regional anesthesia is an option when attempting to avoid general anesthesia, but it does come with limitations. Patients who are young, restless, or uncooperative may not be ideal candidates for a procedure that utilizes regional anesthesia as opposed to general anesthesia [15].

Most dermatologic procedures are performed using local anesthesia for a variety of reasons. It has been shown that general anesthesia, when the patient is completely sedated, paralyzed, and intubated, carries a greater risk of adverse events than local anesthesia [16]. General anesthesia is typically reserved for more invasive dermatological procedures or circumstances that require sedation. It may be necessary for MMS that requires the removal of multiple lesions, deep excisions, or complex reconstructions [17]. General anesthesia may be considered for patients who suffer from extreme anxiety or a phobia of medical procedures [17]. It is frequently necessary for pediatric cases due to the limited ability of the patient to cooperate during the procedure [18]. The literature shows that, while general anesthesia for elective pediatric dermatology cases carries a low risk when managed by a skilled anesthesiologist, its use in adult patients is significantly more costly and associated with higher complication rates [16, 17]. The decision to use general anesthesia for a Mohs procedure should be made on a case-by-case basis, considering the patient’s individual needs and the nature of the procedure.

In dermatologic procedures, it is a common technique to inject local anesthetic directly into the wound to minimize patient discomfort. There are three stages of wound healing: inflammation, granulation, and remodeling. Local anesthetics have been shown to disrupt the first and second stages of wound healing [19]. Despite this, some studies have shown no long-term alteration in wound healing with the addition of local anesthetic because the strength of the wounds is normal [20]. Studies have also evaluated the effect of topical anesthetics on wound healing in mice, and they show no significant hindrance to wound healing [21]. 

It has been shown that patients who receive regional anesthesia peri-operatively to control post-operative pain have reduced tissue Po2. Neutrophils depend on subcutaneous Po2 to kill infectious organisms. Therefore, pain control might increase the patient’s risk of infection [22]. It was also previously stated that local anesthetics inhibit the first two stages of wound healing, the first being inflammation. Local anesthetic injection into a patient’s wound has an anti-inflammatory effect that could increase the risk of infection [23]. Local anesthesia adequately improves patients’ post-operative experiences by diminishing incision-associated pain, but the physician must consider the unfavorable effects that accompany their use (Table 1).

**Table 1:** Local anesthetics commonly used in dermatological procedures.

Local Anesthetic	Common Concentrations	Onset of Action	Duration of Action	Common Dermatological Procedures	Special Considerations
Lidocaine	1% or 2%, with or without epinephrine	1-3 minutes	1-2 hours without epinephrine, 2-4 hours with epinephrine	Excisional biopsies, Mohs surgery, cryosurgery	Epinephrine prolongs effect and reduces bleeding; avoid in areas with poor circulation (e.g., fingers, nose) [15]
Bupivacaine	0.25% or 0.5%	5-10 minutes	4-8 hours	Long procedures, nerve blocks	Longer duration, good for post-op pain; higher risk of systemic toxicity [15]
Mepivacaine	1% or 2%	3-5 minutes	1.5-2 hours	Minor excisions, dermatologic surgeries without epinephrine	Less vasodilation compared to lidocaine; can be used without epinephrine [15]
Prilocaine	0.5% to 1% (often in EMLA cream with lidocaine)	2-5 minutes	1-2 hours	Topical anesthetic for minor dermatologic procedures	Risk of methemoglobinemia at higher doses [15]
Tetracaine	4% topical or 0.5% injection	15-20 minutes	2-3 hours	Minor skin surgeries, laser treatments	Long onset, strong potency for topical use [15]
Ropivacaine	0.5% or 0.75%	5-15 minutes	3-6 hours	Nerve blocks, large excisions	Similar to bupivacaine, but with less cardiotoxicity [15]

Anesthesia-related complications in dermatology

Allergic Reactions

Although relatively uncommon, allergic reactions to anesthetic agents in dermatologic procedures are significant due to their potential severity. Local anesthetics, such as lidocaine and bupivacaine, have been known to trigger hypersensitivity reactions ranging from mild skin rashes to severe anaphylactic shock. The incidence of these allergic reactions is relatively low, with estimates suggesting they occur in less than 1% of patients [24]. Identifying an allergic reaction typically involves a thorough patient history, assessment of clinical symptoms, and, if necessary, allergy testing. Effective management includes discontinuing the offending anesthetic immediately, providing symptomatic relief, and administering emergency treatments like epinephrine and supportive care when needed [24]. Proactive preoperative screening and educating patients about the signs of potential allergic reactions are crucial to reducing these risks.

Local Anesthetic Systemic Toxicity (LAST)

Systemic toxicity from local anesthetics, especially lidocaine, is a severe concern during dermatologic procedures. This toxicity can occur if too much anesthetic is used or if there's an accidental injection into a blood vessel, which can raise plasma levels and lead to systemic effects [25]. Symptoms of toxicity include central nervous system disturbances such as tinnitus and seizures, as well as cardiovascular issues like arrhythmias [26]. Lower concentrations of anesthetics and aspirating before injection can further reduce the risk of systemic toxicity [27]. While high doses of local anesthetic are generally avoided due to the risk of systemic toxicity, there are certain circumstances where they may be necessary. For example, extensive procedures covering large areas of the body will require a higher dose of local anesthetic to achieve adequate pain control. If the procedure is expected to be lengthy, a higher dose may be used to ensure the analgesic effects last the entire duration of the procedure. In emergency situations, local anesthetics are often combined with epinephrine or antifibrinolytic drugs to control bleeding or hemorrhage [28]. Patients may have specific conditions that require a larger dose of local anesthesia, such as patients with hypersensitivity to pain or resistance to local anesthetics. Varying patient factors such as age (children and elderly), sex, pregnancy, concurrent drug use, and other comorbidities such as hepatic impairment and cardiovascular disease may affect the absorption of local anesthetics into the body [29,30]. Individuals with red hair have been found to require a higher dose of anesthetic to achieve the same level of analgesia as individuals of other hair colors, possibly due to a genetic variation in their MC1R gene [31]. While these situations may warrant higher doses of local anesthetic, it is crucial to weigh the potential benefits against the risks of systemic toxicity. Healthcare providers should carefully consider the patient’s individual factors and the specific procedure before administering a higher dose of local anesthetic. Additionally, providers should calculate doses based on the patient's weight and the anesthetic volume used and monitor the patient closely throughout the procedure [27].

Delayed Healing and Scarring

Anesthesia-related factors can sometimes lead to delayed wound healing and abnormal scarring after dermatologic procedures. Delayed healing may be due to compromised local blood flow, tissue damage, or the effects of the anesthetic agents on cellular processes [32]. Additionally, abnormal scarring, including hypertrophic scars and keloids, can be influenced by the type of anesthetic used, its method of administration, and individual patient characteristics [33]. To minimize these risks, it's important to use optimal wound care techniques, avoid unnecessary tissue trauma, and consider adjunctive treatments like silicone gel sheeting or corticosteroid injections to manage scarring [34].

Special considerations in anesthesia for dermatological procedures

Pediatric and Geriatric Populations

Anesthesia for pediatric and geriatric patients requires a tailored approach due to their unique physiological and pharmacological differences. For pediatric patients, challenges include managing smaller and more susceptible airways, necessitating precise airway management and appropriately sized endotracheal tubes. Children also have higher metabolic rates and less mature organ systems, which affect how anesthetic drugs are processed. Thus, dosing needs to be adjusted carefully to ensure safety and effectiveness. Additionally, given the varying levels of cooperation among children, using age-appropriate sedation strategies, such as preoperative counseling and distraction techniques, can help minimize the need for higher doses of sedatives. Continuous monitoring is essential to detect any adverse effects early [35].

For elderly patients, anesthesia management must account for age-related changes, such as decreased organ function and diminished physiological reserve, which affect drug metabolism and clearance. Older adults often have reduced hepatic and renal function, which requires adjustments in anesthetic dosing and careful selection of agents. They may also be more sensitive to anesthetics, with increased risks of cognitive impairment and prolonged sedation. Therefore, a cautious approach involving lower doses and possibly regional anesthesia or carefully titrated intravenous agents is recommended [36]. Collaborating with geriatric specialists can optimize care, ensuring comprehensive preoperative assessments and vigilant postoperative monitoring [9].

Patients With Comorbidities

Managing anesthesia for patients with comorbid conditions, such as diabetes or cardiovascular disease, presents additional challenges. Diabetes can impair wound healing and heighten the risk of infection, while cardiovascular issues may complicate blood pressure and heart rate management during procedures. Anesthetic protocols for these patients should involve thorough preoperative evaluations, modifications in anesthetic agents, and close monitoring during the procedure to address any potential complications [9]. Coordination with other healthcare professionals and considering the patient’s overall health status are crucial for optimizing outcomes [37].

Psychological Impact

Addressing the psychological impact of anesthesia in dermatologic procedures is vital for improving patient experience and outcomes. Anxiety about anesthesia and the procedure itself can significantly affect patient satisfaction and perceived pain levels [38]. This anxiety can result in heightened stress responses and discomfort, potentially complicating both the procedure and recovery. Preoperative counseling is crucial, providing patients with clear information about what to expect before, during, and after the procedure. Detailed explanations about the anesthesia process, potential side effects, and recovery can help alleviate preoperative anxiety and give patients a sense of control.

Additionally, using anxiolytics or sedatives can help manage anxiety and enhance comfort, leading to a more relaxed and cooperative demeanor during the procedure [39]. Effective sedation techniques improve patient comfort and facilitate smoother procedures by reducing movement and stress. This can enhance procedural precision and lead to better outcomes and a more efficient recovery. Managing anxiety effectively can also improve overall patient satisfaction and contribute to a more positive perception of the care received [38]. By integrating strategies like preoperative counseling, appropriate use of anxiolytics, and careful sedation management, the patient experience in dermatologic procedures can be significantly enhanced, resulting in better outcomes and higher satisfaction levels.

Innovations in anesthesia for dermatology

Advances in Anesthetic Formulations and Delivery

New and emerging topical anesthetics have significantly improved the comfort of dermatological procedures. Topical lidocaine is commonly used in dermatology due to its easy application and low risk of adverse effects. Although effective in reducing discomfort and mild pain during dermatological procedures, healthcare providers must consider the skin's barrier properties and the short duration of action of lidocaine. Research has been directed toward enhancing efficacy and increasing patient satisfaction by developing new drug formulations and delivery systems. Some emerging advances in anesthetic formulations and drug delivery involve the use of nanotechnology, microneedle patches, and laser therapy. Precision medicine is rapidly growing and enables healthcare providers to tailor anesthetic regimens to individual patients, resulting in better clinical outcomes.

Nanotechnology

Anesthetic nanocarriers are nanoparticles composed of natural synthetic/semisynthetic polymers, lipids, or other materials that are used to encapsulate anesthetic drugs to improve their effects [40]. The small size of nanoparticles allows them to effectively penetrate the stratum corneum, the most superficial layer of skin that acts as a barrier for the passage of foreign materials [41]. Depending on their use, nanoparticles can be customized to alter the penetrance of the skin layers, remaining more superficial for topical drugs or penetrating deeper through the skin into the blood vessels for transdermal delivery. Nanoparticle systems can improve topical anesthetic penetrance in the skin without increasing unwanted systematic transdermal transport in outpatient dermatological procedures [42]. The most prevalent types of nanoparticles currently used for cutaneous drug delivery are liposomal nanoparticles, microemulsions (MEs), and polymeric nanoparticles [41]. Nanoparticles containing fixed drug combinations may allow the penetrance of compounds and their synergistic effects into the skin [43].

Liposomes and MEs

Liposomes are spherical nanoparticles composed of phospholipids that encapsulate anesthetic drugs. Liposome and ME formulations enhance drug solubility and stability, improving efficacy and reducing irritation. MEs are thermodynamically stable drug delivery systems composed of water, oil, and surfactants. The isotropic dispersion of liquids results in ME nanoparticles of controlled size and morphology [44]. Due to their small droplet size, MEs can penetrate the skin barrier better than other drug delivery systems such as creams, ointments, and gels [45]. MEs may contain nonionic, anionic, or cationic surfactants, depending on their intended application. The composition, charge, and structure of surfactants allow for the customization of drug properties such as penetrance, solubility, and bioavailability [46]. Surfactants reduce the interfacial tension between water molecules and lipophilic molecules in the stratum corneum, which disrupts the skin barrier and furthers skin permeation [47]. MEs enhance skin adhesion and penetration of drugs, allowing them to be used in various dermatological settings. An ex-vivo study found that a depot lidocaine-loaded ME may enhance and prolong the effects of local anesthesia [48].

Microneedle Patches

Microneedle patches are a novel drug delivery system that utilizes tiny needles to penetrate the skin and deliver drugs directly into underlying tissues. Microneedle patches used in combination with anesthetics can increase drug absorption and provide sustained anesthesia. These patches offer several advantages over traditional drug delivery methods, particularly in dermatology. Lidocaine HCl encapsulated dissolving microneedles (LiDMNs) delivered sufficient drug concentrations to maintain anesthesia with a faster onset time and longer residual time than traditional topical cream applications or injections [49]. Microneedle-assisted lidocaine treatment was able to accelerate the onset of action from 40 to 10 minutes, substantially enhancing the efficacy of localized anesthesia [50]. Rapidly separable bubble microneedle patches are a form of microneedle patch that quickly penetrates the skin due to the sheer force of administration, resulting in a convenient and rapid method of delivering anesthetic drugs [51]. The use of a microneedle patch in combination with lidocaine yielded significantly smaller visual analogue scale (VAS) and verbal rating scale (VRS) pain scores compared to the use of lidocaine alone [52].

Minimally Invasive Anesthetic Techniques

Minimally invasive anesthesia refers to techniques that minimize the physical trauma and discomfort associated with traditional anesthesia methods. These approaches aim to provide adequate pain relief while reducing the risk of complications and improving patient recovery. Prioritizing patient comfort through pain and anxiety reduction has been demonstrated to enhance clinical outcomes, foster stronger physician-patient relationships, and boost overall patient satisfaction [53]. The choice of minimally invasive anesthesia will depend on the type of procedure, the patient’s medical history, and patient preferences. While minimally invasive anesthesia techniques are generally safe, there are potential risks associated with any medical procedure, and these should be appropriately relayed to the patient so that they may make informed decisions about their healthcare.

Cryoanesthesia: Cryoanesthesia is a technique that involves applying extreme cold temperatures (-70°F to -180°F) to the skin to induce numbness and reduce pain through nerve receptor desensitization [11]. This method is often used in dermatological procedures to numb the skin immediately before minor treatments or surgery. While research is exploring various modalities for use in cryoanesthesia, even a simple ice application has proven to be as effective as 20% benzocaine gel [54]. In the past, mediums such as ethyl chloride and liquid nitrogen have been used to cause a transient drop in local skin temperatures. However, they were associated with adverse effects related to prolonged freezing, such as frostbite, dyspigmentation, and atrophic scarring [55-57]. Cryoanesthesia with ethyl chloride was significantly more effective at alleviating pain compared to topical 5% lidocaine gel [56]. CryoVIVE® is a newly introduced cryoanesthesia device that performs targeted cooling of the epidermis using CO2 gas. CryoVIVE® devices have thermo-sensor-enabled real-time temperature and duration monitoring to prevent the adverse effects associated with traditional forms of cryoanesthesia. CryoVIVE® was demonstrated to reduce pain by 40% without any observed adverse effects [58].

Laser therapy: Medical lasers are devices that use a focused beam of light to treat or remove tissue. Laser therapy has become a popular and effective treatment option for various skin conditions, including lesion removal, as it is considered less painful than traditional methods.

Personalized anesthesia: Precision medicine is a personalized approach to healthcare that tailors medical treatments to each patient's specific needs and characteristics [59]. By analyzing an individual’s genetic makeup, lifestyle, and environmental factors, healthcare providers can develop highly personalized treatment plans that optimize patient care. Genomic analysis uses genetic information to predict individual responses to different anesthetics and tailor treatment plans accordingly. Variations in genes can influence how patients respond to anesthesia. Proteomic analysis analyzes protein biomarkers to identify potential adverse reactions and allergies. Proper investigative studies can provide insight into the appropriate dosage and type of anesthesia needed for a particular patient and aid in predicting potential allergic reactions or adverse side effects. Comprehensive assessments of patient medical history and current medications can help identify individual risk factors and concerns regarding anesthetic choices. The ability to customize drug formulations for specific patient needs and skin types is an area of ongoing research.

Case studies and clinical examples

Case 1: Anesthesia in High-Risk Dermatologic Surgery

Mohs micrographic surgery (MMS) is a precise surgical procedure for treating high-risk skin cancers. MMS is typically performed using local anesthesia, with lidocaine being the most commonly used [60]. Routine monitoring during MMS or other outpatient dermatologic procedures is not currently recommended, but it should be implemented on a case-by-case basis in specific populations at high risk for adverse effects [61]. Dermatologic procedures typically require much less local anesthesia than the maximum recommended dose, making systemic toxicity an uncommon occurrence [62].

Additionally, local anesthetics routinely include epinephrine to extend the duration of anesthesia and decrease systemic absorption and toxicity [63]. Paradoxically, systemic reactions to epinephrine from local anesthetics are reported in 2.0% of patients undergoing MMS (95% CI: 0.1%-3.9%), unrelated to the total dosage of epinephrine administered [64]. Local anesthesia with epinephrine has also been linked with atrial fibrillation in patients with hypertension [61]. In a case report of an 86-year-old man with no history of arrhythmias, an injection of 2 milliliters (mL) of 1% lidocaine plus 1:100,000 epinephrine (0.02 mg) caused tachycardia within two minutes [61]. In this scenario, MMS should be immediately aborted, and intravenous esmolol should be administered [61]. Although rare, healthcare providers should remain alert and aware of various presentations of anesthesia-related complications during dermatologic procedures so that they may quickly recognize and treat the complication. LAST commonly presents with CNS changes, such as agitation, confusion, dizziness, or dysphoria. Prioritizing airway management, circulatory support, and the mitigation of systemic side effects is crucial in the initial treatment of LAST to prevent progression into more severe complications, such as seizures, respiratory arrest, and/or coma [65].

Case 2: Managing Anesthesia-Related Complications in a Dermatology Setting

While rare, the use of anesthesia in a dermatological setting can result in adverse effects, ranging from mild skin irritation to life-threatening anaphylaxis. Most adverse effects can be prevented by performing a thorough medical history intake, allergen testing, and emergency preparedness. Patient medical history can inform healthcare providers if patients have known hypersensitivities to anesthetics, related drugs that may be used during anesthesia, and potential cross-reactivity reactions. Skin prick tests (SPTs) can be performed before the procedure without specialized equipment to determine possible hypersensitivity reactions to local anesthetics [66]. Special care should be taken for at-risk populations.

Contact dermatitis is a minor reaction due to direct contact with local anesthetics. Contact dermatitis presents general symptoms such as pruritus, erythema, and localized edema [67, 68]. Contact dermatitis should resolve upon removal of the irritant [67]. Contact dermatitis should not be confused with actual allergic reactions, type I hypersensitivity reactions mediated by IgE antibodies. Type I hypersensitivity is an immediate reaction characterized by urticaria, angioedema, erythema, pruritus, and flushing, among other symptoms [69]. If a type I hypersensitivity is not immediately recognized, it can rapidly progress to life-threatening anaphylaxis [70]. Type I actual allergic or anaphylactic reactions should be treated with the administration of intramuscular epinephrine and intravenous fluids [71].

Case 3: Cosmetic Outcomes in Dermatologic Surgery With Different Anesthetic Techniques

Aesthetic dermatology is a specialized field that focuses on improving the appearance and health of the skin with medical treatments. Pain management is an integral component of aesthetic dermatology that can affect patient satisfaction and clinical outcomes [72]. The majority of aesthetic procedures performed, such as laser resurfacing, chemical peels, and injections, can be uncomfortable and even painful for some patients. Minimizing pain and anxiety during cosmetic procedures ensures patient comfort and accuracy during injections, as the patient is less likely to be restless or withdraw from the pain sensation. Although there are no statistically significant differences in pain scores among the most commonly used topical anesthetics in cosmetic and laser procedures (2.5% lidocaine/2.5% prilocaine cream (EMLA®), 4% tetracaine, or 4% liposomal lidocaine gel), most patients preferred 4% liposomal lidocaine gel [73].

Future directions and research needs

Gaps in Current Knowledge

Although pain management is a critical component of dermatological procedures, there are still areas where research and development could improve patient outcomes and safety. Longitudinal studies are needed to explore the long-term consequences of different anesthetic modalities used in dermatologic practice. However, this may be challenging, given the rapid emergence of new technologies. Ongoing research should scrutinize and monitor the effects of new anesthesia drugs until more comprehensive studies can be realized. Research can identify proteins involved in anesthetic-induced neurotoxicity and develop strategies to mitigate these effects [74]. The use of traditional anesthetics has been shown to induce neurotoxicity. Therefore, it is crucial to be aware of this complication and investigate the potential long-term effects of all developing anesthetic drugs used in patient populations [75]. With the growing application of nanoparticles in drug delivery, there have been regulatory concerns about their long-term effects on the human body, which necessitates a pressing need to address the shortcomings in toxicology reports of nanoparticles [76]. The small size of nanoparticles may enable access to various organ systems or translocation to unintended areas of the body, necessitating more research to understand the underlying mechanisms of drug targeting [76].

Innovative Research Directions

Nanoparticles: As with everything else in medicine, the field of anesthesia in dermatology is constantly evolving. Emerging research explores pain management options that can improve patient comfort, safety, and outcomes during dermatologic procedures. New materials are continually being studied and tested as nanoparticle carriers for drug delivery. Nanostructured lipid carriers (NLC) are an emerging lipid nanoparticle that may offer advantages over other lipid-based carriers such as solid lipid nanoparticles and liposomes [77]. Cubosomes are lipid-based nanoparticles with potential for active drug targeting via controlled release and ligand-receptor interactions [78]. Recent in vitro, in vivo, and ex vivo animal studies demonstrated that lidocaine-loaded cubosomal gels significantly increased the penetrance of lidocaine into the skin and sustained its release, prolonging its anesthetic effects without any adverse effects [79].

Virtual reality (VR): Distraction techniques may not eliminate pain, but they can help reduce pain perception by focusing patient attention elsewhere and employing different sensory modalities. Distraction techniques are already being used in pediatric settings, such as dental offices, through television or music in exam rooms. Unlike traditional distraction methods, VR is an immersive experience incorporating auditory, visual, and cognitive components. VR may help in pain management through distraction and non-distraction mechanisms by producing neurophysiologic changes related to conditioning and exposure therapies [80]. A meta-analysis found that VR-based interventions may reduce needle-related pain, fear, and anxiety in children and adolescent populations [81]. Further research should determine the variety of ways VR may provide an avenue to distract patients during dermatological procedures, reduce pain, and incorporate informational resources for patient education during procedures. More studies should examine the effect of VR-based interventions in larger patient populations and settings.

## Conclusions

Anesthesia plays a critical role in the success of dermatologic procedures, influencing the patient’s comfort as well as clinical and cosmetic outcomes. This review highlights the wide-ranging effects of different types of anesthesia, including topical, local, regional, and general anesthesia, on wound healing, infection rates, pain management, and the risk of complications such as allergic reactions and systemic toxicity. While local anesthesia remains the standard for most dermatologic interventions due to its efficacy and safety profile, special considerations must be taken when dealing with pediatric, geriatric, and high-risk patients. Despite the progress made in this field, further research is required to better understand the long-term impacts of anesthesia on wound healing and scarring, and to develop safer and more effective anesthetic agents for patients with comorbidities or those at higher risk for complications. Personalized anesthesia, informed by genetic and proteomic analysis, holds great potential to revolutionize patient care by tailoring anesthetic regimens to individual needs, thus optimizing outcomes.
